# Study of Ultra-High Performance Concrete Mechanical Behavior under High Temperatures

**DOI:** 10.3390/ma17174212

**Published:** 2024-08-26

**Authors:** Guilherme S. Sumitomo, Lia L. Pimentel, Ana Elisabete P. G. A. Jacintho, Nadia C. S. Forti

**Affiliations:** Polytechnic School, Pontifical Catholic University of Campinas, Campinas 13086-061, Brazil; lialp@puc-campinas.edu.br (L.L.P.); anajacintho@puc-campinas.edu.br (A.E.P.G.A.J.); nadia.cazarim@puc-campinas.edu.br (N.C.S.F.)

**Keywords:** UHPC, fire resistance, factorial design, compressive strength, elasticity modulus, surface response

## Abstract

The main concern with concrete at high temperatures is loss of strength and explosive spalling, which are more pronounced in high-strength concretes, such as Ultra-High Performance Concrete (UHPC). The use of polymeric fibers in the mixture helps control chipping, increasing porosity and reducing internal water vapor pressure, but their addition can impact its mechanical properties and workability. This study evaluated the physical and mechanical properties of UHPC with metallic and PVA fibers under high temperatures using a 2^3^ central composite factorial design. The consistency of fresh UHPC and the compressive strength and elasticity modulus of hardened UHPC were measured. Above 300 °C, both compressive strength and elasticity modulus decreased drastically. Although the addition of PVA fibers reduced fluidity, it decreased the loss of compressive strength after exposure to high temperatures. The response surface indicates that the ideal mixture—1.65% steel fiber and 0.50% PVA fiber—achieved the highest compressive strength, both at room temperature and at high temperatures. However, PVA fibers did not protect UHPC against explosive spalling at the levels used in this research.

## 1. Introduction

Ultra-High Performance Concrete (UHPC) can be understood as an evolution and combination of the properties of ultra-high strength concrete (because it reaches values of compressive strength above 150 MPa and tensile strength in bending above 6 MPa), self-compacting concrete (because it has high fluidity), and fiber-reinforced concrete (because it has fibers in its composition). Moreover, it also has high durability due to its dense microstructure and very low porosity [1,2,3,4,5].

Despite presenting a high initial cost and excessive environmental footprint due to its need for large quantities of cement and superplasticizer in its composition, it allows structural elements to have a smaller cross-section to meet the requested efforts and, therefore, less material needed for production, as well as increasing the useful life of the structure, thus requiring less maintenance. Added to this, more sustainable solutions for the development of UHPC are being studied, such as the use of supplementary cementitious materials and substituting aggregates for industrial waste powder [5,6]. Ref. [7] carried out a study of sustainable UHPC using Al_2_O_3_ micro powder in its composition, evaluating its mechanical properties using microscopy, and also analyzing the environmental and economic impact. As a result, they obtained a UHPC that, in addition to having an improvement in workability, compressive and flexural strengths, and elasticity modulus, presented a reduction of 11.43% in energy consumption, 7.01% in CO_2_ emissions, and 1.18% in production costs when compared to conventional UHPC.

UHPC has a wide area of applications, some of which are structural elements in bridges and walkways, architectural works such as facades and roof panels, and reinforcement and structural restoration [3]. However, these applications may present a risk of being exposed to high-temperature situations, such as a fire [8]. Even though concrete is a non-combustible material with low thermal conductivity, such exposure to fire ends up causing a drop in its structural performance, in addition to possible damage such as cracks and spalling that can compromise the entire structure. Concretes with high strengths (such as UHPC) are more susceptible to explosive spalling, a phenomenon that is theoretically caused by the pressure of water vapor present in the internal pores of the composite [9].

As UHPC is a fiber-reinforced material, its behavior during heating may vary depending on the type of fiber used in its production. The reinforcement of steel fibers provides better performance in terms of its mechanical properties, allowing it greater resistance against the stresses generated during the heating process [10,11]. The use of polymeric fibers is considered a means of preventing spalling because polymeric fibers have a melting temperature below 300 °C (some below 200 °C, depending on their type) and, after melting, create a network of pores within the concrete that becomes a means of escape for trapped water vapor [12]. However, there are not many studies about the behavior of UHPC under high temperatures or the use of polymeric fibers in its composition.

Constitutive modeling is an approach that aims to describe specific behaviors of a material, creating mathematical models that are used to simulate structures under different conditions. However, using existing models for conventional and/or high-strength concrete to predict the behavior of UHPC is not effective due to the complex influence between its constituent materials, thus making it a field with few related studies [13,14,15].

In order to overcome this research gap, computational models are being studied and developed, modeling complex problems that involve nonlinear relationships between input and response variables. Refs. [13,14] developed a constitutive stress–strain model for UHPC confined with normal-strength steel and high-strength steel. Through optimization of the hyperparameters of the base and secondary models, a database of stress–strain curves for confined UHPC was generated, which served as training for the constitutive model based on machine learning, resulting in high predictive accuracy for stress–strain curves of confined UHPC. Ref. [15] employed in their study numerical models to create a database for bridge columns made with UHPC, which subsequently formulated a predictive model using machine learning to establish a correlation between engineering demand parameters and parameters that define column geometry, material properties, and internal reinforcement, thus proposing a model that presents strong predictive performance in various damage states for bridge columns made with UHPC.

The objective of this work is to evaluate the fluidity of UHPC in the fresh state as well as its resistance to compression and elasticity modulus when subjected to high temperatures when the concrete is produced with steel and PVA fibers in different contents through the construction of response surfaces generated by factorial planning that was used as a constitutive model.

## 2. Materials and Methods

### 2.1. Composition of UHPC

The UHPFRC mix developed by Ref. [16] (shown in Table 1) was chosen for use as they obtained compressive strength with cubic specimens subjected to curing in water at room temperature, varying between 137 MPa and 175 MPa at 28 days, being compatible with the class UHPFRC 150/165 specified by Ref. [17].

**Figure 1:**
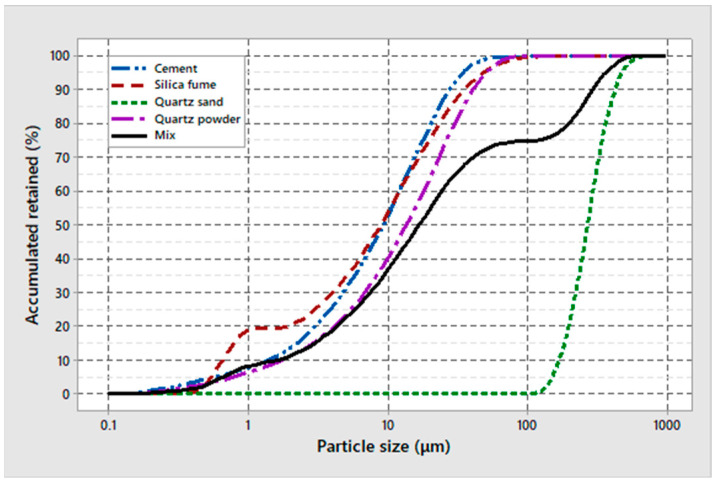
Granulometric curve of the materials.

The materials used in this research were chosen to have similar characteristics to the materials used by Ref. [16]. The cement used for this study was CPV ARI according to Ref. [18], the sand and quartz powder were produced by an ore processing company, therefore having controlled granulometry, and the silica fume used was Centrilit Fume S, dispersed in an aqueous solution. The granulometric curves of these materials, obtained by laser granulometry assay, are shown in Figure 1. The water used came from the public distribution system, and the additive used was based on polycarboxylate, MC-Powerflow 4001 by MC-Bauchemie (São Paulo, Brazil).

The metallic fibers used are steel fiber DRAMIX OL 13/.20 by ArcelorMittal (São Paulo, Brazil) with a length of 13 mm, a diameter of 0.20 mm, and a tensile strength of 2160 MPa. The polymeric fibers used are PVA fiber branded KURALON (RCS15) by Kuraray (São Paulo, Brazil) with 12 mm in length, 0.04 mm in diameter, and a tensile strength of 1560 MPa.

### 2.2. Factorial Design

A factorial design is represented by N^k^, where k is the number of factors under study and N is the number of levels for each factor. In this type of planning, all possible combinations of the levels existing in each factor are investigated, and the influence of the factors on the response variables of interest is quantitatively evaluated [19,20].

**Figure 2:**
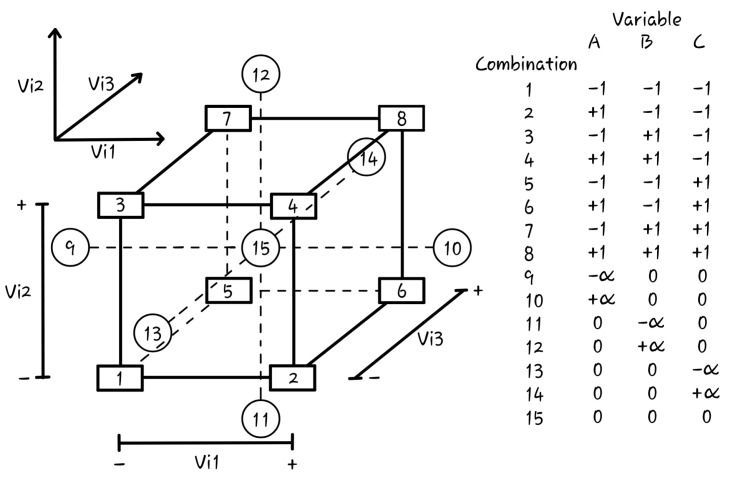
Factorial design 2^3^.

Factorial design at two levels (2^k^) is indicated for the quantification of the influence of each factor on the response since, in addition to providing a smaller number of observations, it allows for the construction of linear or quadratic models in the factors to describe the studied system [20,21,22]. The Central Composite Rotational Design One is a procedure used to optimize the system. This type of design is symmetrical and second-order, presenting 2^k^ factorial points (a combination of levels represented as “−” or “−1” for the minimum value and “+” or “+1” for the maximum value), one or more central points (represented as “0”), and 2 × k axial points (represented as ±α, with α equal to 2^(k/4)^) [21]. As shown in Figure 2, it is possible to see, for example, the graphical representation of the factorial design 2^3^ and its combinations.

The mathematical model used to generate the response surface represents the relationship between the response function and the factor levels and can be a first-order (linear) or second-order (quadratic) model, the latter being presented by Equation (1) [19,21,23].

(1) $$\gamma =\beta_{0}+\sum_{i=1}^{K}\beta_{i}X_{i}+\sum_{i=1}^{K}\beta_{ii}X_{i}^{2}+\sum_{i=1}^{K-1}\sum_{i=2_{i<j}}^{k}\beta_{ij}X_{i}X_{j}+\varepsilon$$

where X_1_, X_2_…, X_K_ are the independent variables that influence the response Y, β_0_ is the intercept (displacement) coefficient, β_1_, β_2_… β_k_ are the linear coefficients (first-order), β_11_, β_22_…, β_kk_ are the quadratic coefficients (second-order), β_1j_, β_2j_…, β_kj_ are the interaction coefficients, and ε is the experimental error.

Although factorial design is not widely used in civil engineering, it can be used to develop new products, optimize processes, and reduce costs by enabling a reduction in the number of experiments and verifying, analyzing, and quantifying the effects of the variables under study in a systematic and statistically solid way. For instance, Ref. [24] used a two-level factorial design to study the interaction effect of fiber dosage, steel reinforcement, and plate thickness on the punching shear strength of fiber-reinforced concrete flat slabs. Ref. [25] determined and analyzed the mechanical and durability properties of three optimized concrete mixtures incorporating glass powder and gold mine tailings using two-level factorial designs with a central composite design.

### 2.3. Combination of Study Factors

In order to study the influence of steel and PVA fibers on the mechanical behavior of UHPC when subjected to high temperatures, which in this case are compressive strength and elasticity modulus, three factors were determined: temperature in °C, amount of steel fiber, and amount of PVA fiber, both in % of volume. In this way, the planning method adopted was the central rotational composite design: 2^3^ + two central points + six axial points. Since this model requires the least number of experiments to be carried out and, therefore, less consumption of materials and less use of laboratories, given the limited amount of materials available to be used in this work and the restricted use of laboratories due to the COVID-19 pandemic, this method is suitable for this research.

The software used for the data processing was STATISTICA 7, and Table 2 shows the variables determined for this study as well as their value ranges.

Combinations of variable levels were determined by Ref. [26] through the definition of the coded levels presented in Table 3, with the repetition of two central points, as shown in Table 4.

The range of values for PVA fibers was selected based on experimental studies by Refs. [27,28], who replaced steel fiber with PVA fiber at 0.25% and 0.50% by volume and observed that there was no spalling when subjected to high temperatures. Ref. [29] also observed an improvement in the mechanical behavior.

However, experimental studies carried out by Refs. [12,27,29] show that replacing steel fibers with polymeric ones causes a drop in their compressive strength, which is highlighted as the replacement dosage increases when the UHPC is subjected to high temperatures, as noted in this research.

In the case of steel fibers, the determined value range considers the possibility of studying the response surface in the case of replacement of up to 0.50% of the steel fibers (the maximum value of the interval of PVA fibers) and also in the case of PVA fiber additions, keeping the dosage of 2.00% of steel fibers constant.

Finally, to study the mechanical behavior as the temperature increases, the ambient temperature (20 °C) was adopted as the initial temperature, and it was raised to 600 °C since, based on Refs. [9,30,31], reaching temperatures close to 600 °C, the concrete begins to lose its ability to resist the load, a situation in which the compressive strength and elasticity modulus of the concrete can reach values below half of those verified before being subjected to high temperatures.

### 2.4. Mixing and Casting Procedure

The mixing of materials was carried out using a high-intensity vertical mixer from the brand Perfecta with a capacity of up to 10 L and with three speeds: low (95 rpm), medium (180 rpm), and high (280 rpm). The adopted procedure consists of the following steps:

(1) Add 50% of the cement + 100% of the silica + 100% of the water, and mix for 5 min at low speed;
(2) Add 25% of the cement + 100% of the additive, and mix for 5 min at medium speed;
(3) Add 25% of the cement and mix for 5 min at high speed;
(4) Add 100% of the sand + 100% of the quartz powder, and mix for 5 min at high speed;
(5) Add fibers evenly and mix for 5 min, with the speed being low during fiber insertion and high soon after.

Once the materials are mixed, the molds are completely filled in a single layer, and the consolidation is carried out using a vibrating table for one minute. For the curing procedure, the specimens were kept submerged in water with lime at ambient temperature. Cylindrical specimens (ϕ75 × 150 mm) were molded.

### 2.5. Description of the Drying Process and Application of High Temperatures

After 28 days of immersion curing, the specimens were dried at ambient temperature for 24 h and oven-dried at 60 °C for 48 h. They were removed from the oven and chilled to ambient temperature, after which they were placed in a muffle furnace. Initiating heating from ambient temperature to the specified temperature, as determined by the factorial design method and indicated in Table 1, the temperature was kept constant for one hour, and then the muffle was turned off and kept closed so that the specimens cooled down slowly, thus avoiding thermal shock. The specimens were tested under compression the day after the end of heating.

### 2.6. Tests Performed

Soon after the completion of the material mixing, the consistence index of the UHPC in the fresh state was determined using a rigid mold with a conical trunk shape with a base diameter, top diameter, and height equal to, respectively, 125 mm ± 0.5 mm, 80 mm ± 0.5 mm, and 65 mm ± 0.5 mm, according to Ref. [32]. The mold was filled in a single layer, and after the top was scraped, the mold was removed vertically without the process of lifting and dropping the table. After 2 min, the largest and smallest diameters were determined, and the fluidity was obtained through the average of these two values.

The compressive strength test was carried out in accordance with Ref. [33] using three specimens of dimensions ϕ75 × 150 mm for each series of UHPC at 28 days. The elasticity modulus test was carried out in accordance with Ref. [34] using two specimens of dimensions ϕ75 × 150 mm for each series of UHPC at 28 days.

## 3. Results and Discussion

### 3.1. Consistence Index

The results of the consistence index (flow table) are presented in Table 5, and the consistence obtained allows for classifying each mixture according to Ref. [17]:

1. Ca: UHPC, which may be self-compacting (diameter 270 mm). Generally able to be placed without vibration or use of a mechanical flow aid;
2. Cv: viscous UHPC (230 mm diameter < 270 mm). Generally able to be placed without vibration but which requires the use of a mechanical flow aid;
3. Ct: UHPC exhibiting a flow threshold (diameter < 230 mm). Generally able to flow under the effect of dynamic shearing but whose free surface at rest may keep sloping.

**Figure 3:**
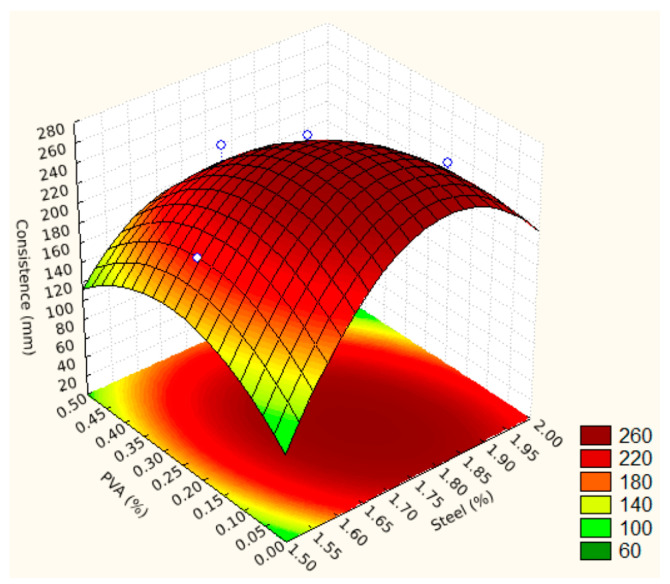
Response surface for the consistence index. The blue circles are results obtained in the tests.

For a better visualization of the fluidity behavior of the UHPC in its fresh state, the 2^2^ factorial design was used, resulting in a model presented by Equation (2) and the response surface presented in Figure 3.

D = −5210.0499444322 + 6028.525299141 × A − 1663.3333333329 × A^2^ + 1611.1337948474 × P − 883.33333333313 × P^2^ − 751.11111111085 × A × P (2)

where D is the consistence index, in mm; A is the dosage of steel fibers, in %; and P is the dosage of PVA fibers, in %.

The generated response surface has a value of R^2^ = 0.85446 and has a critical point when the percentage of steel is equal to 1.78% and the percentage of PVA is equal to 0.16%, with the maximum fluidity calculated from 271.91 mm. It is possible to observe that the PVA fibers generate a decrease in fluidity as a function of the increase in their dosage.

According to Ref. [35], the fibers generate an interlock in the fresh state, providing fluidity loss, and the greater the fiber volume, the greater this loss. Unlike what was expected, for the lowest possible dosage of steel fibers and PVA, the samples showed lower values of fluidity.

It is also possible to observe that the end regions of the response surface, with the exception of the 0.00% PVA and 2.00% steel end, present values below the diameter of the mold base used in the test (125 mm ± 0.5 mm), which is inconsistent since it is not possible for the UHPC in its fresh state to decrease its initial diameter once the mold is removed.

### 3.2. Compressive Strength and Elasticity Modulus

**Figure 4:**
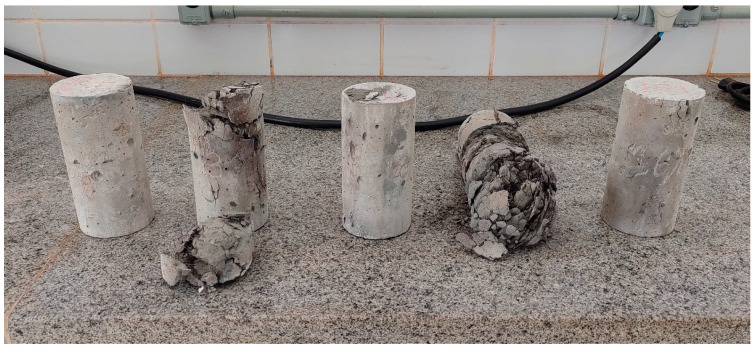
Spalling occurred during heating.

During the heating process of the specimens described in Section 2.5, spalling occurred in some mixtures (illustrated in Figure 4) when the temperature exceeded 300 °C and, consequently, it was not possible to obtain the value of the compressive strength.

In order to achieve better data processing using the STATISTICA 7 software, the values of compressive strength and elasticity modulus were estimated through a succession of factorial designs, using the data obtained in the tests and adopting second-order mathematical models.

1. A factorial 2^3^ design was used to determine an initial response surface;
2. The response surface equation from the previous step was used to determine the f_c_ values of the missing cells to carry out two 2^2^ factorial designs, wherein the PVA fiber was set at 0.25% and the steel fiber was set at 1.75%. In this way, the f_c_ values were determined for a temperature equal to 310 °C;
3. Once the f_c_ values for a temperature equal to 310 °C were obtained, a 2^3^ factorial design was carried out in order to determine the remaining f_c_ values (for temperatures equal to 482 °C and 600 °C);
4. Finally, a final 2^3^ factorial design was performed using all tests and estimated values to construct the response surface to be analyzed.

Using MANOVA at a significance level of 5%, it was possible to check whether the adjusted model is well adjusted or not to meet the null hypothesis that the generated response surface is flat. Using the F value, it was possible to determine whether the null hypothesis was accepted (when F_calculated_ < F_table_) or rejected (when F_calculated_ > F_table_).

#### 3.2.1. Compressive Strength

The mathematical model obtained by Ref. [26] for compressive strength is presented in Equation (3), with R^2^ equal to 0.99457. Table 6 presents, for each trace, the compressive strength values obtained in the laboratory and estimated by the model.

f_c_* = −1513.6719920032 + 0.0883464082 × T − 0.0002067771 × T^2^ + 1849.9247355629 × A − 505.4942376002 × A^2^ + 678.7990434340 × P + 0.0017623997 × P^2^ − 0.0000006460 × T × P − 367.5148148148 × A × P(3)

where f_c_* is compressive strength; A is the dosage of steel fibers, in %; P is the dosage of PVA fibers, in %; and T is the exposure temperature, in °C. Developed by Ref. [26], Table 7 and Table 8 show, respectively, the MANOVA factors and MANOVA table.

Table 7 shows that only the variables PVA (for the quadratic model), the interaction between Temperature and Steel, and the interaction between Temperature and PVA are not significant, with their *p* values equal to 1.000. The other variables are statistically significant, presenting large magnitudes, with the exception of the variable Steel (linear model), which presents the lowest intensity, being closer to the limit that represents the value *p* = 0.05.

Based on Table 8, the null hypothesis is rejected, as the F_calculated_ value is greater than the F_table_ value and, therefore, the response surface will present some curvature.

**Figure 5:**
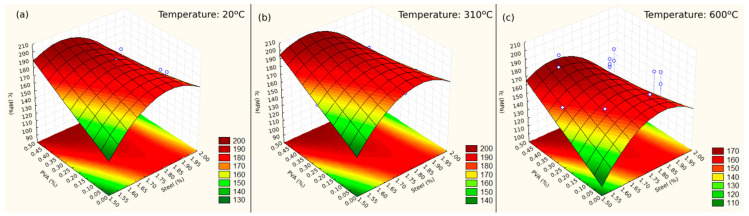
Response surfaces for: (**a**) 20 °C, (**b**) 310 °C, and (**c**) 600 °C. The blue circles are results obtained in the tests.

The values obtained by the model are very close to the results obtained in the laboratory and the estimated values and are therefore appropriate to describe the data. Three response surfaces (Figure 5) were constructed to verify the behavior of the compressive strength of the UHPC at 20 °C (Figure 5a), 310 °C (Figure 5b), and 600 °C (Figure 5c) against the variation in the dosages of steel fibers and PVA.

The compressive strength tends to increase with the addition of more PVA fibers to the UHPC mix. However, for steel fiber amounts exceeding 1.90%, this trend is reversed. When examining the response surface, compressive strength exhibits a parabolic behavior with steel fiber dosage, and the lowest values stay at the extremes.

In the case studied, the region with the highest compressive strength values for the analyzed temperatures falls within PVA dosages ranging from 0.35% to 0.50% and steel fiber dosages below 1.90%. The dosage that yields the maximum compressive strength is 0.50% for PVA and 1.65% for steel fiber.

Considering the addition of PVA fibers to a mix with a constant steel fiber dosage of 2.00% (commonly used), any amount of PVA fiber added reduces the compressive strength. Comparing mix 2 with 3 and mix 6 with 7, which present the same temperatures and total fiber dosage equal to 2.00% of each other but with different percentages of replacement of steel fiber by PVA fiber, it is noted that, in the first case, there is an increase in compressive strength of 7.62% and, in the second, there is an increase of 8.23%. This illustrates that replacing steel fiber with PVA fiber, in addition to providing greater prevention of spalling, does not generate a large variation in its resistance.

Analyzing the effect of elevated temperatures on UHPC, an increase in ambient temperature up to 310 °C caused a low variation in compressive strength, with an increase below 5%. However, for higher temperatures, the resistance drop is clear, which is between 10% and 20% compared to the resistance obtained at ambient temperature. Based on Refs. [9,30,31], the reduction in compressive strength at higher temperatures is caused by the deterioration of the cement paste. In the range of 300 °C and 600 °C, carbonation and expansion of the matrix pores occur, as do the decomposition of hydrated calcium silicate (CSH) and CaCO_3_, forming CaO. As a result, there is a reduction in volume and an increase in cracks, and the concrete loses its load-bearing capacity.

Table 9 presents the compressive strength results of UHPC with steel and PVA fibers obtained by Refs. [15,27].

Analyzing the results predicted by the factorial design obtained by Equation (3) for the same fiber dosages used by these authors, the values found for the conventional dosage of UHPC (2.00% of steel fiber) were very close to those obtained experimentally by Ref. [27], with the difference between the values less than 10%. However, when analyzing the UHPC with a dosage of 1.50% steel fiber and 0.50% PVA fiber, the difference increases, mainly above 400 °C, probably due to the difference in heating speed and time of, respectively, 5 °C/min and 3 h adopted in the studies.

For dosages that are outside the range established in this research (Section 2.3), the compressive strength values found with the model are inconsistent.

When comparing with Ref. [12], the values found by the design for ambient temperature (20 °C) were between 15% and 20% lower. This difference may have occurred due to the type of curing that the specimens were subjected to, since Ref. [12] applied thermal curing, which consisted of subjecting the specimens to curing immersed in water at a temperature of 90 °C for two days. After exposure to temperatures close to 1000 °C, the values predicted by the model were between 23% and 56% higher than those obtained by Ref. [12]. Such a difference may have been generated by the loss of model accuracy since the temperature value is outside the studied range.

#### 3.2.2. Elasticity Modulus

The mathematical model obtained by Ref. [26] for elasticity modulus is presented in Equation (3), with R^2^ equal to 0.99805. Table 10 presents, for each trace, the compressive strength values obtained in the laboratory and estimated by the model.

E* = 38.6743290512 + 0.1399743729 × T − 0.0003268141 × T^2^ + 1.4122540478 × A − 0.0003231396 × A^2^ + 84.7319703553 × P + 220,5596768604 × P^2^ − 0.0000000311 × T × A − 0.2717441860 × T × P − 75.77777778 × A × P(4)

where E* is elasticity modulus; A is the dosage of steel fibers, in %; P is the dosage of PVA fibers, in %; and T is the exposure temperature, in °C. Developed by Ref. [26], Table 11 and Table 12 show, respectively, the MANOVA factors and MANOVA table.

Table 11 shows that only the variables Steel (for the quadratic model) and the interaction between Steel and Temperature are not significant. Observing the value of F_calculated_, it is evident that the Temperature variable (for the linear model) has the greatest magnitude among the other factors. It is possible that the cause of this is due to the lack of data obtained in experiments with elevated temperatures. For other variables in other cases of effects and interaction effects, they appear to be statistically significant, being below *p* = 0.05.

Based on Table 12, the null hypothesis is rejected as the F_calculated_ value is greater than the F_table_ value and, therefore, the response surface will show some curvature.

**Figure 6:**
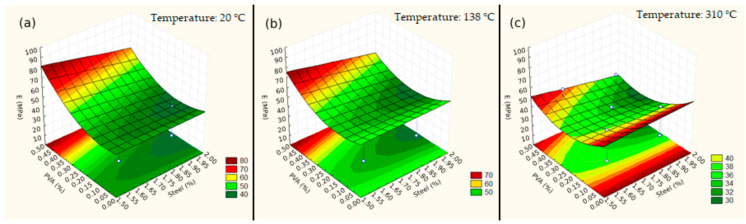
Response surfaces for: (**a**) 20 °C, (**b**) 138 °C, and (**c**) 310 °C. The blue circles are results obtained in the tests.

The values obtained by the model are very close to the results obtained in the laboratory and the estimated values, making them appropriate for describing the data. Three response surfaces were constructed (Figure 6) to verify the behavior of the elasticity modulus of the UHPFRC at a given temperature (20 °C, 138 °C, and 310 °C) in relation to varying dosages of steel fibers and PVA. However, as no results were obtained for values above 310 °C, graphs were not constructed for temperatures of 482 °C and 600 °C, as in the case of compressive strength.

The elasticity modulus predicted by the model obtained becomes equivocal when observing higher temperature values due to the lack of experimental data for temperatures above 310 °C, thus finding negative values as shown in Table 8, which does not match reality. This fact may have occurred due to the sharp drop in the modulus for higher dosages of PVA as the exposure temperature increases, thus causing the model to predict very low values for higher temperatures.

In Table 13, it is possible to observe from the results obtained by Refs. [27,36] that there was a drop in the elasticity modulus as the temperature increased, but it is still possible to obtain results for higher temperatures.

## 4. Conclusions

The present work investigated the consistence index of UHPC in its fresh state and its compressive strength for mixtures containing steel and PVA fibers at high temperatures, analyzing the response surface of a factorial design at two levels (2^k^).

Regarding fluidity, the quantity of fibers in the UHPC mix leads to a decrease, with PVA fibers being those that generated the greatest reduction. A very large quantity of fibers in the mixture impairs its workability and, consequently, its densification, and may thus impair its mechanical properties.

It is observed in the response surfaces for the compressive strength and elasticity modulus that the UHPC suffers a reduction in its properties as a function of the temperature increase.

When PVA fibers are used, the loss of strength is more pronounced because UHPC develops more voids after PVA fibers reach their melting temperature, contributing to a further reduction in compressive strength. Considering the function of PVA fibers to prevent spalling, they were not able to prevent all cases that occurred, especially at more extreme temperatures.

The blending of PVA fibers with steel fibers had a positive influence on the mechanical properties, highlighting the greater quantities of PVA fibers that generated the highest results for both compressive strength and elasticity modulus. The best use of the two fibers occurred when there was a constant dosage of fibers, with the partial substitution of steel fibers for PVA fibers.

Observing both the behavior of compressive strength and the elasticity modulus, the dosage of fibers in the UHPC that improves both properties under the conditions presented in this study would be 1.65% steel fiber and 0.50% PVA, regardless of the temperature range analyzed.

The extreme regions of the surfaces proved to be more imprecise, given the results of fluidity (which obtained flow table values smaller than the mold’s initial diameter), compressive strength (obtaining distant values when compared to other authors), and elasticity modulus (negative values for temperatures of 600 °C), therefore not being suitable for predicting its behavior for values outside the treated range. This occurs because they cover the most extreme values of the adopted data range, being further away from the experimental data and thus losing their precision. Added to this, the occurrence of spalling harmed the development of the compressive resistance response surfaces and, mainly, the elasticity modulus, whose analysis was limited to temperatures up to 310 °C.

In this study, three variables were selected that influence both compressive strength and elasticity modulus. As a suggestion for future work, there are other factors, such as types of materials and void index, that can be added to the factorial planning to make it more comprehensive, in addition to studying different intervals of the factors raised in this study to make the most precise response surface, especially at the edges.

## Figures and Tables

**Table 1:** UHPFRC mix design (kg/m^3^).

Cement	Silica Fume	Quartz Sand	Quartz Powder	Superplasticizer	Water
922.880	179.160	1015.168	92.288	36.915	179.160

**Table 2:** Variables and value range utilized.

Variable	Symbol	Type	Value Range	
			Minimum	Maximum
Temperature (°C)	T	Independent	20	600
Steel fiber (%)	FA	Independent	1.5	2.0
PVA fiber (%)	FPVA	Independent	0	0.5
Fluidity (mm)	Vr1	Dependent	-	-
Compressive strength (MPa)	Vr2	Dependent	-	-
Elasticity modulus (GPa)	Vr3	Dependent	-	-

**Table 3:** Coded levels.

Variable	Coded Variable				
	−α	−1	0	1	α
T	20	138	310	482	600
FA	1.50	1.60	1.75	1.90	2.00
FPVA	0.00	0.10	0.25	0.40	0.50

**Table 4:** Combination of coded variables and identification of mixtures by Ref. [26].

	Mix	Coded Variable			Experimental Values		
		T	FA	FPVA	T (°C)	FA (%)	FPVA (%)
Factorial points	T1	−1	−1	−1	138	1.60	0.10
	T2	−1	−1	1	138	1.60	0.40
	T3	−1	1	−1	138	1.90	0.10
	T4	−1	1	1	138	1.90	0.40
	T5	1	−1	−1	482	1.60	0.10
	T6	1	−1	1	482	1.60	0.40
	T7	1	1	−1	482	1.90	0.10
	T8	1	1	1	482	1.90	0.40
Axial points	T9	−1.682	0	0	20	1.75	0.25
	T10	1.682	0	0	600	1.75	0.25
	T11	0	−1.682	0	310	1.50	0.25
	T12	0	1.682	0	310	2.00	0.25
	T13	0	0	−1.682	310	1.75	0.00
	T14	0	0	1.682	310	1.75	0.50
Center points	T15	0	0	0	310	1.75	0.25
	T16	0	0	0	310	1.75	0.25

**Table 5:** Consistence index.

Mix	Consistence Index (Flow Table)	
	Diameter (mm)	Classes (NF P-18 470 [17])
1 and 5	203.33	Ct
2 and 6	175.40	Ct
3 and 7	257.93	Cv
4 and 8	162.40	Ct
9	278.05	Ca
10	251.70	Cv
11	204.45	Ct
12	191.23	Ct
13	250.80	Cv
14	215.08	Ct
15 and 16	328.53	Ca

**Table 6:** Compressive strength obtained in the model (f_c_*) and in the laboratory (f_c_) [26].

Mix	Temp. (°C)	Steel (%)	PVA (%)	Resp. Surface f_c_* (MPa)	Experimental f_c_ (MPa)
1	138	1.60	0.10	169.86	169.47
2	138	1.60	0.40	196.70	196.71
3	138	1.90	0.10	182.78	182.66
4	138	1.90	0.40	177.13	176.81
5 ^#^	482	1.60	0.10	156.15	155.76
6 ^#^	482	1.60	0.40	182.99	183.00
7 ^#^	482	1.90	0.10	169.07	168.95
8 ^#^	482	1.90	0.40	163.42	163.10
9	20	1.75	0.25	186.22	186.22
10	600	1.75	0.25	163.10	163.10
11 ^#^	310	1.50	0.25	163.25	163.25
12 ^#^	310	2.00	0.25	157.66	157.66
13 ^#^	310	1.75	0.00	183.14	229.29
14 ^#^	310	1.75	0.50	200.96	249.91
15	310	1.75	0.25	192.05	195.00
16	310	1.75	0.25	192.05	189.10

^#^ mix with the estimated compressive strength.

**Table 7:** MANOVA factors for compressive strength [26].

Factors	Sum of Squares (SS)	Degree of Freedom (df)	Mean Square (MS)	F_calculated_	*p*
(1) Temperature (°C) (L)	643.130	1	643.130	221.45	0.000
Temperature (°C) (Q)	315.872	1	315.872	121.16	0.000
(2) Steel (%) (L)	38.150	1	38.150	13.14	0.011
Steel (%) (Q)	1168.896	1	1168.896	402.50	0.000
(3) PVA (%) (L)	387.631	1	387.631	133.48	0.000
PVA (%) (Q)	0.000	1	0.000	0.00	1.000
1 L by 2 L	0.000	1	0.000	0.00	1.000
1 L by 3 L	0.000	1	0.000	0.00	1.000
2 L by 3 L	547.022	1	547.022	188.36	0.000
Error	17.425	6	2.904	-	-
Total SS	3144.827	15	-	-	-

**Table 8:** MANOVA table for compressive strength [26].

	SS	df	MS	F_calculated_	F_table_
Regression (SSR)	3190.222	9	354.469	122.06	4.10
Residual	17.425	6	2.904	-	-
Total (SST)	3207.646	15	-	-	-

**Table 9:** Compressive strength (in MPa) obtained in the studies [15,27] for different temperatures.

Author	Steel Fiber (%)	PVA Fiber (%)	20 °C	100 °C	200 °C	300 °C	400 °C	500 °C	600 °C	700 °C	1049 °C
Sanchayan and Foster (2016) [27]	2.00	0.00	170.00	176.15	188.68	172.87	-	-	-	-	-
	1.50	0.50	150.00	158.88	183.06	187.50	143.59	87.34	80.92	41.94	-
	1.00	1.00	158.00	163.20	170.46	161.12	141.89	50.93	48.51	30.14	-
	0.50	1.50	145.00	143.57	163.60	150.72	117.81	87.29	59.62	31.00	-
	0.00	2.00	134.00	146.34	152.07	134.88	92.57	72.29	-	-	-
Park et al. (2019) [15]	1.50	0.20	192.00	-	-	-	-	-	-	-	11.80
	1.50	0.30	194.00	-	-	-	-	-	-	-	17.40
Equation (3)	2.00	0.00	165.88	170.97	173.60	172.09	-	-	-	-	-
	1.50	0.50	189.30	194.38	197.02	195.51	189.87	180.10	166.19	148.14	-
	1.00	1.00	143.73	148.81	151.44	149.94	144.30	134.52	120.61	102.57	-
	0.50	1.50	29.17	34.25	36.88	35.38	29.74	19.96	6.05	−11.99	-
	0.00	2.00	−154.38	−149.30	−146.67	−148.17	−153.81	−163.58	-	-	-
	1.50	0.20	151.04	-	-	-	-	-	-	-	14.50
	1.50	0.30	163.80	-	-	-	-	-	-	-	27.25

**Table 10:** Elasticity modulus obtained in the model (E*) and in the laboratory (E) [26].

Mix	Temp. (°C)	Steel (%)	PVA (%)	Resp. Surface E* (GPa)	Experimental E (GPa)
1	138	1.60	0.10	48.78	48.83
2	138	1.60	0.40	59.53	59.71
3	138	1.90	0.10	46.92	46.98
4	138	1.90	0.40	50.97	51.04
5 ^#^	482	1.60	0.10	17.76	17.93
6 ^#^	482	1.60	0.40	0.72	-
7 ^#^	482	1.90	0.10	15.89	16.08
8 ^#^	482	1.90	0.40	−7.85	-
9	20	1.75	0.25	44.27	44.27
10 ^#^	600	1.75	0.25	−31.47	-
11 ^#^	310	1.50	0.25	38.27	38.27
12 ^#^	310	2.00	0.25	29.50	29.50
13	310	1.75	0.00	53.13	53.13
14	310	1.75	0.50	42.21	42.21
15	310	1.75	0.25	33.89	36.91
16	310	1.75	0.25	33.89	30.86

^#^ mix with the estimated elasticity modulus.

**Table 11:** MANOVA factors for elasticity modulus [26].

Factors	Sum of Squares (SS)	Degree of freedom (df)	Mean Square (MS)	F_calculated_	*p*
(1) Temperature (°C) (L)	6904.215	1	6904.215	2263.52	0.000
Temperature (°C) (Q)	878.985	1	878.985	288.17	0.000
(2) Steel (%) (L)	93.762	1	93.762	30.74	0.001
Steel (%) (Q)	0.000	1	0.000	0.00	1.000
(3) PVA (%) (L)	145.481	1	145.481	47.70	0.000
PVA (%) (Q)	222.534	1	222.534	72.96	0.000
1 L by 2 L	0.000	1	0.000	0.00	1.000
1 L by 3 L	393.233	1	393.233	128.92	0.000
2 L by 3 L	23.256	1	23.256	7.62	0.033
Error	18.301	6	3.050	-	-
Total SS	9381.874	15	-	-	-

**Table 12:** MANOVA table for elasticity modulus [26].

	SS	df	MS	F_calculated_	F_table_
Regression (SSR)	9363.572	9	1040.397	341.09	4.10
Residual	18.301	6	3.050	-	-
Total (SST)	9381.874	15	-	-	-

**Table 13:** Elasticity modulus results from the studies [27,36].

Author	Steel Fiber (%)	Polymeric Fiber (%)	Elasticity Modulus, in GPa (Exposure Temperature in °C)			
			Room Temperature	20 °C~200 °C	200 °C~400 °C	400 °C~600 °C
Sanchayan and Foster (2016) [27]	2.00	-	46.30	45.92 (200 °C)	43.09 (300 °C)	-
	1.00	1.00 (PVA)	42.10	42.27 (200 °C)	36.94 (300 °C)	6.53 (600 °C)
	-	2.00 (PVA)	40.80	37.30 (200 °C)	33.64 (300 °C)	-
Rasul, M.; Ahmad, S.; Adekunle, S.K.; Al-Dulaijan, S.U.; Maslehuddin, M.; Ali, S.I. [36]	-	0.26 (PP)	47.81	-	45.00 (300 °C)	-
	-	0.52 (PP)	47.25	-	43.13 (300 °C)	-
	-	0.78 (PP)	43.50	-	43.88 (300 °C)	-
	-	1.04 (PP)	46.13	-	39.38 (300 °C)	-

## Data Availability

The data presented in this study are available on request from the corresponding author. The data are not publicly available due to privacy reasons.
